# A comparison of liveweight gain of lambs weaned early onto a herb-clover mixed sward and weaned conventionally onto a ryegrass-clover pasture and herb-clover mixed sward

**DOI:** 10.5713/ajas.18.0301

**Published:** 2018-07-26

**Authors:** W. E. M. L. J. Ekanayake, R. A. Corner-Thomas, L. M. Cranston, P. R. Kenyon, S. T. Morris

**Affiliations:** 1 Sheep Research Centre, School of Agriculture and Environment, Private Bag 11-222, Massey University, Palmerston North, 4442, New Zealand

**Keywords:** Early Weaning, Growth, Herb, Lamb, Pasture Shortage

## Abstract

**Objective:**

The aim of the present study was to identify the impact of early weaning of lambs at approximately seven weeks of age onto a herb-clover mix on the liveweight gain of lambs and their dams.

**Methods:**

In 2015, twin-born lambs that weighed a minimum of 16 kg (n = 134) were randomly allocated to one of three treatments: i) Early-weaned (58 days after the midpoint of lambing) onto an unrestricted allowance (>1,200 kg dry matter/ha) of herb-clover mix (HerbEW); ii) Lambs+dams unweaned onto an unrestricted allowance of herb-clover mix until conventional weaning (95 days after the midpoint of lambing) (HerbCW); iii) Lambs+ dams unweaned onto an unrestricted allowance of grass-clover pasture until conventional weaning (GrassCW). In 2016, twin-born lambs that weighed a minimum of 16 kg (n = 170) were randomly allocated to one of four treatments: i), ii), iii) (similar to 2015) and iv) Lambs+ dams unweaned onto a restricted allowance (<1,200 kg dry matter/ha) of grass-clover pasture until conventional weaning (93 days after the midpoint of lambing) (Restricted-GrassCW).

**Results:**

In 2015, liveweight gain from L58 to L95 of HerbCW and GrassCW lambs did not differ (p>0.05), but were greater than HerbEW lambs. In 2016, HerbCW lambs had greater (p<0.05) liveweight gains from L51 to L93 than GrassCW followed by HerbEW and Restricted-GrassCW lambs. In 2015, liveweight gain from L58 to L95 of HerbEW ewes were greater than both GrassCW and HerbCW ewes while in 2016, liveweight gain of from L51 to L93 GrassCW and HerbCW ewes did not differ (p>0.05) but were greater (p<0.05) than those of HerbEW and Restricted-GrassCW ewes.

**Conclusion:**

These results indicate that when grass-clover pasture supply can be maintained at unrestricted intake level, there may be no benefit of weaning lambs early. However, at restricted pasture conditions lambs can achieve greater liveweight gains when weaned early onto a herb-clover mix.

## INTRODUCTION

A herb-clover mix containing plantain (*Plantago lanceolata*), chicory (*Cichorium intybus*), red clover (*Trifolium pretense*), and white clover (*Trifolium repens*) has been shown to produce greater herbage yields than a predominant perennial ryegrass-white clover pasture in New Zealand [1,2]. Further, the herb-clover mix has higher quality and digestibility than perennial ryegrass-white clover pasture [1,3–5]. These traits of herb-clover mix have resulted in improved ewe performance (liveweight gain, body condition, milk production; [6,7] and pre- and post-weaning lamb growth [1,8,9] compared to a grass-clover pasture.

In lamb production systems in New Zealand, lambs are conventionally weaned between 10 and 14 weeks of age onto grass-clover pastures [10]. Under this system, and especially during seasons where there is a shortage of grass-clover pasture, the ewe and lamb can become competitors for the same feed resource [11]. As a result, the liveweight gain of lambs prior to conventional weaning can be disappointing. In New Zealand, the production of grass-clover pasture can be limited in late-spring and early-summer [12,13]. Therefore, offering *ad-libitum* feeding conditions (>1,200 kg dry matter [DM]/ha; [3,14]) can be a challenge for sheep farmers. Early weaning onto high quality herbages such a herb-clover mix during such conditions is a potential option to reduce overall feed demand, and allow both lambs and ewes to achieve higher liveweight gains [5,11]. To date no studies have examined the use of a herb-clover mix for early weaned lambs. Therefore, the aim of the present study was to identify the impact of early weaning of lambs at approximately seven weeks of age onto a herb-clover mix on the liveweight gain of lambs and their dams. Over a two-year period, unrestricted and restricted pasture conditions were utilized. It was hypothesised that a positive effect for both ewe and her lamb was more likely to occur from early weaning when pasture availability was low.

## MATERIALS AND METHODS

### Experimental design

This study was conducted at Massey University’s Tuapaka farm 15 km east of Palmerston North, New Zealand (latitude 40°20′S, longitude 175°43′E). All manipulations were approved by the Massey University Animal Ethics Committee. Romney ewes (n = 65 in 2015 and n = 83 in 2016) which conceived during a 17-day breeding period and that were diagnosed bearing twin fetuses using transabdominal ultrasound were enrolled in the study. Throughout the gestation period, within each year, ewes were managed as one mob under commercial farming conditions. Lambing began on 02 September in 2015 and 31 August in 2016. All lambs were weighed, ear tagged and identified to their dam within 24 h of birth.

From the midpoint of lambing (L1) until the onset of the study (L58 and L51 in 2015 and 2016, respectively), lambs and ewes were managed as a single mob on a ryegrass dominant clover pasture. Lambs were orally drenched every 28 days, as per standard practice in New Zealand, beginning at L26 in 2015 and L25 in 2016 with Ancare ‘Matrix’ triple combination drench (Merial Ancare, Manukau City, New Zealand) at a rate of 1 mL per 5 kg live weight to reduce the risk of worm burden. Ewes that successfully reared both lambs to minimum of 16 kg were subsequently enrolled in the study and allocated to one of three weaning treatments at L58 in 2015 and four weaning treatments at L51 in 2016 (Table 1). Two grass-clover pasture herbages were used in 2016 to allow for comparison of the impact of early weaning onto herb-clover mix when pasture conditions were either restricted or unrestricted. In 2015, ewes weaned early onto a herb-clover mix (HerbEW) were managed with unweaned ewes and lambs on grass-clover pasture at unrestricted allowance (GrassCW) until conventional weaning. In 2016, ewes in HerbEW treatment were managed with unweaned ewes and lambs on grass-clover pasture at restricted allowance (Restricted-GrassCW) until conventional weaning. Early weaned lambs on the herb-clover mix were managed with unweaned ewes and lambs in both years. Within 1 to 2 h of birth, ewes develop an exclusive bond with their lambs and reject any alien lambs that attempt to suck for the remainder of the lactation [15]. Therefore, it is unlikely that weaned lambs were stealing milk from unweaned ewes.

Three paddocks of herb-clover mix (7.8 ha in total land area) and three paddocks of grass-clover pasture (5.2 ha in total land area) were used for the duration of the study. Lambs and ewes allocated to treatments at L58 in 2015 and L51 in 2016 were rotationally grazed. During the experimental period, pastures were managed to provide *ad-libitum* intakes (>1,200 kg DM/ha) except in the GrassCW treatment in 2016 which was managed to maintain masses below 1,200 kg DM/ha to provide restricted supply of pasture. Ewes and lambs remained on their respective herbages until conventional weaning (at 95 and 93 days from midpoint of lambing in 2015 and 2016, respectively). Lambs and ewes assigned to the HerbEW and HerbCW (Lambs+dams unweaned onto an unrestricted allowance of herb-clover mix until conventional weaning) treatments were gradually introduced to herb-clover mix over a four-day period from L55 in 2015 and from L48 in 2016) of increasing duration on each day (i.e. 4 h day 1, 8 h day 2, 12 h day 3, and 24 h day 4) prior to the onset of the main study. Lambs and ewes were weighed within 1 h of being bought off their herbages on L58, L81 and L95 in 2015 and L51, L82 and L93 in 2016. Post-grazing sward surface heights were maintained by grazing down to a minimum of five cm in the grass-clover pasture and seven cm in the herb-clover mix to provide unrestricted access to herbage. In the Restricted-GrassCW treatment group pasture was maintained below five cm to restrict intakes. In 2015, n = 1 and n = 2 lambs either lost their tag or died in HerbCW and GrassCW treatments, respectively. In 2016, n = 2, n = 4, n = 3, and n = 1 lambs either lost their tag or died in HerbEW, HerbCW, GrassCW, and Restricted-GrassCW treatments, respectively. No ewes died in 2015. In 2016, one ewe either lost her tag or died in HerbEW treatment.

### Herbage measurements

Herbage masses were measured on L58, L81, and L95 in 2015 and, L51, L69, and L82 in 2016. Four random quadrat cuts (0.1 m^2^ each) were taken to ground level from each herbage at each sampling date using an electric shearing hand-piece [16] and samples were oven dried (60°C to 70°C) to a constant weight to estimate herbage mass. In addition, four grab samples per herbage were also collected at each sampling date to estimate the botanical composition and nutritional composition of each herbage [16]. A subsample from each sample was sorted into sown species (Ryegrass, red clover and white clover for grass-clover pasture and plantain red clover and white clover for herb-clover mix), weeds and dead matter, and then oven dried and weighed to attain the botanical composition. The remaining sample was then freeze dried, ground, sieved (one mm) and analysed using *in vitro* methods to determine the nutritional quality; dry matter digestibility (DMD [17]) and crude protein (CP; Dumas’ procedure, AOAC method 968.06 using a Leco total combustion method, LECO Corporation, St. Joseph, MI, USA [18]). Acid detergent fibre (ADF) was analysed by a Tecator Fibretec System [19]. Metabolisable energy content of roughages was calculated using the organic matter digestibility [17].

### Statistical analysis

Live weight and liveweight gain of lambs were subjected to analysis of variance using the MIXED procedure in SAS (Statistical Analysis System, version 9.2; SAS Institute Inc., Cary, NC, USA). The analysis was performed separately for each year due to the differences in the days on which measurements were collected. The effect of treatment on the liveweight gain of lambs and ewes were analysed using individual animal within year as the experimental unit allowing for repeated measures. The effect of weaning treatment on lamb live weight and liveweight gain were analysed using a model that included the fixed effects of weaning treatment and sex of lamb. The exact age of lambs at the start of the treatments was included in the model as a covariate but was found not to be significant (p>0.05), and therefore it was not included in the model. Ewe live weights were analysed in a model including the fixed effects of measurement time, weaning treatment and the interaction between measurement time and weaning treatment. Ewe body condition score was analysed using the GENMOD procedure in SAS that included the fixed effects of weaning treatment.

Botanical composition data were analysed using the MIXED procedure, with a model including the fixed effects of plant species and measurement time. The herbage quality data were analysed using the MIXED procedure, with a model including the fixed effects of pasture type and measurement time. The means were separated using least significant difference (LSD) procedure (p<0.05) in proc general linear model. Herbage masses were analysed using a model that included weaning treatment and measurement times as fixed effects.

## RESULTS

### Botanical composition, herbage mass and nutritional quality of herbage

**Figure 1:**
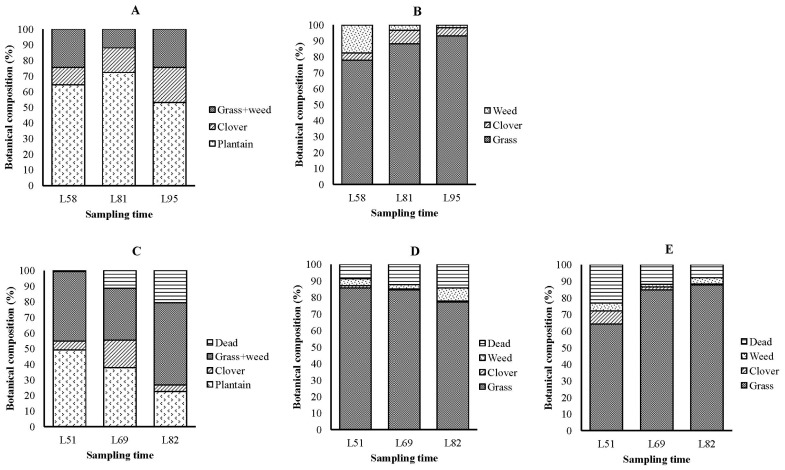
The botanical composition of herbages; herb-clover mix (A), grass-clover pasture (B) in 2015 and herb-clover mix (C), grass-clover pasture (D) and restricted-grass-clover pasture (E) in 2016. L, days after the midpoint of lambing; on herbage A and C there were HerbEW lambs, HerbCW lambs, and their ewes. On herbage B there were GrassCW lambs and their ewes and HerbEW ewes. On herbage D there were GrassCW lambs and their ewes. On herbage E there were Restricted-GrassCW lambs and their ewes and HerbEW ewes. HerbEW, early weaning onto unrestricted allowance of herb-clover mix; HerbCW, lambs+dams offered an unrestricted allowance of herb-clover mix until conventional weaning; GrassCW, lambs+dams offered unrestricted allowance of grass-clover pasture until conventional weaning; Restricted-GrassCW, lambs+dams offered with restricted allowance of grass-clover pasture until conventional weaning.

The mean percentage of plantain in herb-clover mix was 63.3% ±5.6% and 36.5%±7.7% in 2015 and 2016, respectively (Figure 1). Mean percentage of total clover in herb-clover mix was 16.4%±3.3% in 2015 and 9.2%±4.3% in 2016. The remaining portion of the herb-clover mix was made up of grasses and weeds. In both years, ryegrass was the dominant species (77% to 93% in 2015 and 64% to 87% in 2016) in the grass-clover pasture (Figure 1). The mean percentage of clover in grass-clover pasture was 6.1%±1.2% in 2015 and 1.0%±0.2% in 2016. The remaining portion of the grass-clover pasture was made up of weeds.

In 2015 the herbage mass of unrestricted herb-clover mix and grass-clover pasture were maintained at a minimum of 2,647±349 and 1,352±349 kg DM/ha, respectively. In 2016, the herbage mass was maintained at a minimum of 3,501±275 for unrestricted herb-clover mix and 1,353±390 kg DM/ha for grass-clover pasture. The minimum and maximum masses of restricted grass-clover pasture in 2016 were 799±390 and 935±318 kg DM/ha, respectively.

In 2015, the CP content of grass-clover pasture at the start of the study was greater (p<0.05) than that of herb-clover mix (Table 2). At L95, however the reverse was observed and no difference (p>0.05) was observed at L81. The ADF content of grass-clover pasture and herb-clover mix at L58 and L81 did not differ (p>0.05), but at L95 grass-clover pasture had a greater (p<0.05) ADF content than the herb-clover mix. DMD of herb-clover mix was greater (p<0.05) than that of grass-clover pasture at all three measurement times. The metabolisable energy (ME) content of grass-clover pasture was greater (p< 0.05) than herb-clover mix at L58 but reverse was observed at L81 and L95.

In 2016, at the start of the study the CP content of herb-clover mix and restricted-grass-clover pasture did not differ (p>0.05) but was less (p<0.05) than that of grass-clover pasture (Table 2). At L69 and L82, the CP content of restricted-grass-clover pasture, grass-clover pasture and herb-clover mix differed (p<0.05). At L51, the ADF content of grass-clover pasture and herb-clover mix did not differ (p>0.05) but was less (p<0.05) than that of restricted-grass-clover pasture. At L69, the ADF content of herb-clover mix did not differ (p> 0.05) from the ADF content of grass-clover pasture but was greater (p<0.05) than that of restricted-grass-clover pasture. At L82, the ADF content of herb-clover did not differ (p>0.05) from the ADF content of restricted-grass-clover pasture but was greater (p<0.05) than grass-clover pasture. At L51, DMD of herb-clover mix was greater (p<0.05) than that of grass-clover pasture which in turn was greater (p<0.05) than that of restricted-grass-clover pasture. At L69, DMD of herbage did not differ (p>0.05) between the three treatments. At L82, DMD of grass-clover pasture and restricted-grass-clover pasture did not differ (p>0.05) but was greater (p<0.05) than that of herb-clover mix. At the start of the study (L51), the ME content of all treatments differed (p>0.05). The ME content of herb-clover mix was greater (p<0.05) than that of grass-clover pasture followed by restricted-grass-clover pasture. At L69, the ME content of herb-clover mix and restricted-grass-clover pasture did not differ (p>0.05) but was greater (p<0.05) than grass-clover pasture. At the end of the study the ME did not differ (p>0.05) between herbages.

### Lamb live weight and liveweight gain

In 2015, the live weight of lambs at L81 and L95 in HerbCW and GrassCW treatment groups did not differ (p>0.05) but were greater (p<0.05) than lambs in HerbEW (Table 3). The liveweight gain of lambs between L58 and L95 in the HerbCW (325±7 g/d) and GrassCW (321±7 g/d) treatments did not differ (p>0.05) but were greater (p<0.05) than those of HerbEW lambs (251±7 g/d).

In 2016, the live weight of lambs in HerbCW treatment group at L82 and L93 was greater (p<0.05) than lambs in HerbEW, GrassCW, and Restricted-GrassCW treatment groups (Table 3). Live weight at L82 and L93 of HerbEW and GrassCW lambs did not differ (p>0.05) but were greater (p<0.05) than those of Restricted-GrassCW lambs. Lamb liveweight gains between L51 and L93 of HerbCW (307±8 g/d) lambs was greater (p<0.05) than those of GrassCW (263±7 g/d), HerbEW (240±7 g/d) and Restricted-GrassCW (153±7 g/d) lambs.

### Ewe live weight, liveweight gain, and body condition score

In 2015, at L58 the live weight of ewes in each treatment group did not differ (p>0.05) (Table 4). At L81, live weight of ewes in HerbEW did not differ (p>0.05) from ewes in GrassCW, but they were heavier (p<0.05) than HerbCW ewes. At L95, HerbEW ewes were heavier (p<0.05) than both HerbCW and GrassCW ewes. Liveweight gain between L58 and L95 of HerbEW ewes (211±18 g/d) were greater (p<0.05) than both GrassCW (142±18 g/d) and HerbCW ewes (130±19 g/d).

In 2016, live weight of ewes at L51 did not differ (p>0.05) (Table 4). Live weight of ewes in HerbCW and GrassCW treatment groups at L82 and L93 did not differ (p>0.05) but were heavier (p<0.05) than HerbEW and Restricted-GrassCW ewes. Liveweight gain between L51 and L93 of GrassCW (65±21 g/d) and HerbCW (61±20 g/d) ewes did not differ (p>0.05) but were greater (p<0.05) than those of HerbEW (−35±20 g/d) and Restricted-GrassCW (−102±20 g/d) ewes. In both years, body condition score of ewes in each treatment group did not differ (p>0.05) (Table 5).

## DISCUSSION

This study aimed to identify the impact of early weaning of lambs approximately 51 to 58 days after the midpoint of lambing using a herb-clover mix. Early weaned ewes were managed with unweaned ewes and lambs in grass-clover mix until conventional weaning age (in 2015 on unrestricted grass-clover pasture and in 2016 on restricted-grass-clover pasture). In the New Zealand system during late spring to early summer it is unlikely farmers would choose to graze weaned ewes on a high value herbage as a mechanism for them to gain body condition. In contrast lambs are given the priority to achieve high liveweight gains to allow for an earlier slaughter date. Therefore, in this study early weaned lambs were managed with unweaned ewes and lambs on herb-clover mix in both years.

In 2015, when both herbages allowed for unrestricted allowance (>1,200 kg DM/ha) early weaning onto herb-clover mix resulted slower growth rates of lambs compared to those weaned at a conventional age on the herb-clover mix and grass-clover pasture. However, pasture supply is often restricted during late spring and early summer in New Zealand [12,13], therefore, in 2016 an additional weaning treatment; restricted allowance of grass-clover pasture (<1,200 kg DM/ha) was added to simulate this scenario. In 2016, lambs weaned early onto the herb-clover mix had greater liveweight gains than lambs left with their lambs on restricted grass-clover pasture. Restricted pasture availability during late lactation is known to have negative impacts on milk yield [20], length of lactation [21], and lamb liveweight gain [22]. Therefore, early weaning onto alternative forages such as a herb-clover mix can be a useful management tool if the availability of grass-clover pasture is likely to result in the ewe and lamb becoming competitors [11] resulting in restricted intake. It is important to note that in both years, growth of early weaned lambs on unrestricted allowance of pasture to conventional weaning was greater than 240 g/d. Therefore, even in years in which pasture availability was not limiting early weaning onto a herb-clover mix can be utilised to achieve acceptable lamb growth rates. The slower growth of early weaned lambs onto herb-clover mix compared to lambs kept with their dams on herb-clover mix until conventional weaning is likely explained by lack of milk intake and the lamb’s inability to compensate for this. It is also reported that ewes grazing a herb-clover mix showed an increased milk production [8].

The current study design also allowed for a comparison of the growth of lambs weaned conventionally on unrestricted herb-clover and grass-clover pasture. Previous studies have shown that lambs on a herb-clover mix have heavier weights than lambs on grass-clover mix at conventional weaning [8]. In this study, there was no difference in weaning weights of lambs at conventional weaning in 2015 but in 2016 lambs on herb-clover mix were heavier than on grass-clover pasture. A potential explanation for this could be herbage quality and composition between years. In general, studies that have reported greater growth of lambs post weaning onto herb-clover mix, the ME of herb-clover mix was clearly greater than that of the grass-clover pasture [1,23]. However, in this study, the ME of herb-clover mix and grass-clover pasture did not differ always and when they did, the differences were small. The clover content of grass-clover pasture was higher in 2015 than in 2016. While in 2016 the grass-clover pasture had some dead plant matter that was not present in 2015 and only 1% clover. Lambs are known to selectively graze higher ME plant components (white clover) amongst a grass-clover pasture [24,25], and achieve greater liveweight gains when grazed as a pure clover sward [26]. Given lower clover content in grass-clover pasture in 2016 than in 2015, it would have been difficult for lambs to select white clover, which has higher digestibility and ME, and gain live weights in 2016. Combined these compositional changes could help explain why there was no difference of lamb growth in 2015 in herb-clover mix and grass-clover pasture but there was in 2016.

Early weaning of lambs was advantageous for the ewe in 2015 but this was not found to be the case in 2016. The difference of ewe liveweight between years was likely due to the feed offered to ewes post weaning in each year. In 2015, weaned ewes were offered unrestricted grass-clover pasture while in 2016 they were offered restricted grass-clover pasture post weaning. Nonetheless, no differences in ewe body condition score were observed in both years. Combined these results suggest that early weaning can be used as a tool to increase ewe liveweight gain if subsequently the ewes are offered unrestricted pasture conditions. However, the loss of live weight per day on the restricted conditions in 2016 was not as large as the live weight per day of unweaned ewes under same feeding conditions. This is likely due to the removal of lactational nutritional requirement from weaned ewes. This indicates that under conditions where the grass-clover pasture supply is restricted early weaning can still be advantageous for the ewe.

## CONCLUSION

The effect of early weaning onto herb-clover mix on lamb liveweight gain was more apparent when pasture conditions were restricted. This suggests that lambs can be weaned early onto herb-clover mix to gain greater live weight under conditions where grass-clover pasture supply is restricted than unrestricted. Early weaning of lambs onto herb-clover mix can also have positive effect on ewe liveweight gain particularly under unrestricted supply of grass-clover pasture conditions. Combined these results indicate that the lambs most likely to benefit from early weaning onto a herb-clover mix are those that are grazing with their dams on restricted grass-clover pastures.

## Figures and Tables

**Table 1:** Summary of the experimental design including the number of lambs allocated to each weaning treatments

Weaning treatment1)	Number of lambs	Treatment description
2015		
HerbEW^A^	44	Early weaning at 58 after the mid-point if lambing (L58) with unrestricted allowance of herb-clover mix (plantain, red clover and white clover) until conventional weaning at L95
HerbCW^B^	42	Lambs+dams offered an unrestricted allowance of herb-clover mix until conventional weaning at L95
GrassCW^C^	44	Lambs+dams offered unrestricted allowance of grass-clover pasture (Ryegrass, red clover and white clover) until conventional weaning at L95
2016		
HerbEW^D^	44	Early weaning at L51with unrestricted allowance of herb-clover mix until conventional weaning at L93
HerbCW^E^	42	Lambs+dams offered an unrestricted allowance of herb-clover mix until conventional weaning at L93
GrassCW^F^	38	Lambs+dams offered unrestricted allowance of grass-clover pasture until conventional weaning at L93
Restricted-GrassCW^G^	40	Lambs+dams offered with restricted allowance of grass-clover pasture until conventional weaning at L93

EW, early weaning at 58 and 51 days after the midpoint of lambing in 2015 and 2016, respectively; CW, conventional weaning at 95 and 93 days after the midpoint of lambing in 2015 and 2016, respectively; Unrestricted allowance, >1,200 kg dry matter/ha; Restricted allowance, <1,200 kg dry matter/ha.

1) Early weaned ewes in A were managed with unweaned ewes and lambs in C in 2015 until conventional weaning. Early weaned ewes in D were managed with unweaned ewes and lambs in G in 2016 until conventional weaning. Early weaned lambs in A and D were managed with unweaned ewes and lambs in B and E in 2015 and 2016, respectively.

**Table 2:** Nutritional composition of herbages (least-squares mean±SEM)

Herbage1)		CP (%)	ADF (%)	DMD (%)	ME (MJ/kg)
2015					
Herb-clover mix^A^	L58	15.9^b^±1.1	19.9^a^±1.1	73.0^e^±0.5	10.1^c^±0.1
	L81	14.7^b^±1.1	31.0^c^±1.1	69.2^c^±0.5	10.1^c^±0.1
	L95	15.0^b^±1.1	23.3^b^±1.1	73.0^e^±0.5	10.7^e^±0.1
Grass-clover pasture^B^	L58	22.6^c^±1.1	21.4^ab^±1.1	71.0^d^±0.5	10.4^d^±0.1
	L81	15.3^b^±1.1	32.5^c^±1.1	64.4^b^±0.5	9.7^b^±0.1
	L95	12.1^a^±1.1	35.0^d^±1.1	59.5^a^±0.5	8.9^a^±0.1
2016					
Herb-clover mix^C^	L51	16.4^b^±1.2	25.5^a^±2.5	70.0^e^±1.0	10.2^e^±0.3
	L69	13.1^a^±1.2	37.6^d^±2.5	63.3abc±1.0	9.1^d^±0.3
	L82	12.8^a^±1.2	38.2^d^±2.5	62.0^a^±1.0	8.5bcd±0.3
Grass-clover pasture^D^	L51	20.2^cd^±1.2	28.3^ab^±2.5	66.1^d^±1.0	9.1^d^±0.3
	L69	17.9^bc^±1.2	35.6^cd^±2.5	62.5^ab^±1.0	8.2^ab^±0.3
	L82	16.4^b^±1.2	27.5^ab^±2.5	64.2bcd±1.0	9.0^cd^±0.3
Restricted-Grass-clover pasture^E^	L51	16.4^b^±1.2	34.7^cd^±2.9	61.7^a^±1.0	7.5^a^±0.3
	L69	20.6^d^±1.2	31.0^bc^±2.5	63.2abc±1.0	8.5bcd±0.3
	L82	19.6^cd^±1.2	38.3^d^±2.5	64.6^cd^±1.0	8.2abc±0.3

SEM, standard error of the mean; CP, crude protein; ADF, acid detergent fibre; DMD, dry matter digestibility; ME, metabolisable energy content; L, days after the midpoint of lambing.

1) On herbage A and C there were HerbEW lambs and HerbCW lambs and their ewes. On herbage B there were GrassCW lambs and their ewes and HerbEW ewes. On herbage D there were GrassCW lambs and their ewes. On herbage E there were Restricted-GrassCW lambs and their ewes and HerbEW ewes.

a–e Means with different superscripts are significantly different within each year (p<0.05).

**Table 3:** Effect of weaning treatment on live weight of lambs (least-squares mean±SEM)

Weaning treatment1)	n	Live weight (kg)	n	Live weight (kg)	n	Live weight (kg)
2015		L58		L81		L95
HerbEW^A^	44	22.5±0.3^a^	43	27.8±0.3^b^	44	31.8±0.3^d^
HerbCW^B^	42	22.5±0.3^a^	42	29.8±0.3^c^	41	34.5±0.3^e^
GrassCW^C^	44	22.7±0.3^a^	42	30.0±0.3^c^	42	34.2±0.3^e^
2016		L51		L82		L93
HerbEW^D^	44	19.9±0.3^a^	43	28.4±0.3^d^	42	30.0±0.3^e^
HerbCW^E^	42	20.0±0.3^a^	39	30.5±0.3^e^	38	33.0±0.3^f^
GrassCW^F^	38	19.7±0.3^a^	38	28.5±0.3^d^	35	30.8±0.3^e^
Restricted-GrassCW^G^	40	19.5±0.3^a^	39	24.8±0.3^b^	39	25.8±0.3^c^

SEM, standard error of the mean; L, days after the midpoint of lambing; HerbEW, early weaning onto unrestricted allowance of herb-clover mix; HerbCW, lambs+dams offered an unrestricted allowance of herb-clover mix until conventional weaning; GrassCW, lambs+dams offered unrestricted allowance of grass-clover pasture until conventional weaning; Restricted-GrassCW, lambs+dams offered with restricted allowance of grass-clover pasture until conventional weaning.

1) Lambs in A and D grazed with lambs and their ewes in B and E in 2015 and 2016, respectively. Lambs in B and E grazed with their ewes in B (2015) and E (2016). In 2015, lambs in C grazed with C ewes and A ewes. In 2016, F lambs grazed with their ewes in F. Lambs in G grazed with G ewes and D ewes.

a–f Means with different superscripts are significantly different within each year (p<0.05).

**Table 4:** Effect of weaning treatment on live weight of ewes (least-squares mean±SEM)

Weaning treatment1)	n	Live weight (kg)	n	Live weight (kg)	n	Live weight (kg)
2015		L58		L81		L95
HerbEW^A^	22	72.0±1.8^a^	21	77.1±1.8^de^	22	79.8±1.8^e^
HerbCW^B^	21	70.8±1.7^a^	20	72.6±1.7abc	21	75.5±1.7bcd
GrassCW^C^	22	70.6±1.7^a^	22	73.7±1.7abcd	22	75.9±1.7^cd^
2016		L51		L82		L93
HerbEW^D^	22	70.8±1.6^b^	22	68.8±1.6^ab^	21	69.5±1.6^ab^
HerbCW^E^	21	72.3±1.9bcd	19	75.4±1.9^d^	21	74.8±1.9^cd^
GrassCW^F^	19	69.4±1.8^ab^	19	72.2±1.9bcd	19	72.2±1.9bcd
Restricted-GrassCW^G^	20	71.3±2.0^bc^	20	66.9±2.0^a^	20	67.0±2.0^a^

SEM, standard error of the mean; L, days after the midpoint of lambing; HerbEW, early weaning onto unrestricted allowance of herb-clover mix; HerbCW, lambs+dams offered an unrestricted allowance of herb-clover mix until conventional weaning; GrassCW, lambs+dams offered unrestricted allowance of grass-clover pasture until conventional weaning; Restricted-GrassCW, lambs+dams offered with restricted allowance of grass-clover pasture until conventional weaning.

1) A ewes grazed with C lambs and their ewes in 2015. B and E ewes grazed with their lambs in B and E, respectively. C ewes grazed with C lambs and A ewes in 2015. In 2016, D ewes grazed with G ewes and their lambs. F ewes grazed with their lambs in F. G ewes grazed with their lambs in G and D ewes.

a–d Means with different superscripts are significantly different within each year (p<0.05).

**Table 5:** Effect of weaning treatment on body condition score of ewes (Results displayed as mean with 95% confidence interval)

Weaning treatment1)	n	Body condition score	n	Body condition score	n	Body condition score
2015		L58		L81		L95
HerbEW^A^	22	2.5 (1.9–3.2)	21	2.9 (2.3–3.8)	22	2.9 (2.3–3.7)
HerbCW^B^	21	2.5 (1.9–3.3)	20	2.8 (2.1–3.6)	21	2.7 (2.1–3.5)
GrassCW^C^	22	2.6 (2.0–3.3)	22	2.8 (2.2–3.6)	22	2.7 (2.1–3.5)
2016		L51		L82		L93
HerbEW^D^	22	3.0 (2.4–3.8)	22	2.9 (2.3–3.7)	21	3.0 (2.4–3.8)
HerbCW^E^	21	2.9 (2.3–3.7)	19	2.9 (2.3–3.8)	21	3.1 (2.4–3.9)
GrassCW^F^	19	2.8 (2.2–3.6)	19	3.1 (2.3–4.0)	19	2.9 (2.3–3.8)
Restricted-GrassCW^G^	20	2.7 (2.0–3.5)	20	2.6 (1.9–3.4)	20	2.5 (1.9–3.3)

SEM, standard error of the mean; L, days after the midpoint of lambing; HerbEW, early weaning onto unrestricted allowance of herb-clover mix; HerbCW, lambs+dams offered an unrestricted allowance of herb-clover mix until conventional weaning; GrassCW, lambs+dams offered unrestricted allowance of grass-clover pasture until conventional weaning; Restricted-GrassCW, lambs+dams offered with restricted allowance of grass-clover pasture until conventional weaning.

1) A ewes grazed with C lambs and their ewes in 2015. B and E ewes grazed with their lambs in B and E, respectively. C ewes grazed with C lambs and A ewes in 2015. In 2016, D ewes grazed with G ewes and their lambs. F ewes grazed with their lambs in F. G ewes grazed with their lambs in G and D ewes.
